# AT1 and AT2 receptors modulate renal tubular cell necroptosis in angiotensin II-infused renal injury mice

**DOI:** 10.1038/s41598-019-55550-8

**Published:** 2019-12-19

**Authors:** Yongjun Zhu, Hongwang Cui, Jie Lv, Haiqin Liang, Yanping Zheng, Shanzhi Wang, Min Wang, Huanan Wang, Feng Ye

**Affiliations:** 1 Department of Nephrology, The First Affiliated Hospital of Hainan Medical University, Hainan, China; 2 Department of Orthopedics, The First Affiliated Hospital of Hainan Medical University, Hainan, China; 3 The First Clinical College of Hainan Medical University, Hainan, China

**Keywords:** Necroptosis, Chronic kidney disease

## Abstract

Abnormal renin-angiotensin system (RAS) activation plays a critical role in the initiation and progression of chronic kidney disease (CKD) by directly mediating renal tubular cell apoptosis. Our previous study showed that necroptosis may play a more important role than apoptosis in mediating renal tubular cell loss in chronic renal injury rats, but the mechanism involved remains unknown. Here, we investigate whether blocking the angiotensin II type 1 receptor (AT1R) and/or angiotensin II type 2 receptor (AT2R) beneficially alleviates renal tubular cell necroptosis and chronic kidney injury. In an angiotensin II (Ang II)-induced renal injury mouse model, we found that blocking AT1R and AT2R effectively mitigates Ang II-induced increases in necroptotic tubular epithelial cell percentages, necroptosis-related RIP3 and MLKL protein expression, serum creatinine and blood urea nitrogen levels, and tubular damage scores. Furthermore, inhibition of AT1R and AT2R diminishes Ang II-induced necroptosis in HK-2 cells and the AT2 agonist CGP42112A increases the percentage of necroptotic HK-2 cells. In addition, the current study also demonstrates that Losartan and PD123319 effectively mitigated the Ang II-induced increases in Fas and FasL signaling molecule expression. Importantly, disruption of FasL significantly suppressed Ang II-induced increases in necroptotic HK-2 cell percentages, and necroptosis-related proteins. These results suggest that Fas and FasL, as subsequent signaling molecules of AT1R and AT2R, might involve in Ang II-induced necroptosis. Taken together, our results suggest that Ang II-induced necroptosis of renal tubular cell might be involved both AT1R and AT2R and the subsequent expression of Fas, FasL signaling. Thus, AT1R and AT2R might function as critical mediators.

## Introduction

Chronic kidney disease (CKD) is a significant global public health problem, and 14% of the US population and 10.8% of the Chinese population are affected by CKD. Notably, the incidence of CKD is increasing rapidly^1–3^. Much evidence supports the initial injury of renal tubular cells, with the activation of fibroblasts, and their replacement by extracellular matrix as significant factors responsible for the renal injury and remodeling found in CKD^4^. Despite the finding that necroptosis is a more important factor than apoptosis in mediating renal tubular cell injury and tubulointerstitial fibrosis in CKD in rats subjected to subtotal nephrectomy (SNx)^5^, the specific mechanism of activation of this potential pathogenic pathway in renal tubular cells remains unclear.

It is well known that the renin-angiotensin system (RAS), including angiotensinogen, renin, angiotensin-converting enzyme, angiotensin II type 1 receptor (AT1R) and angiotensin II type 2 receptor (AT2R), is activated inappropriately in the diseased kidney during CKD progression. The inappropriate overactivation of the intrarenal RAS plays a critical role in renal tissue injury and remodeling in CKD progression via angiotensin II (Ang II)^6,7^. Importantly, once there is RAS overactivation leading to increased Ang II activity, an insidious stimulatory mechanism causing the sustained activation of the intratubular RAS develops, which can lead to the inappropriate activation of the intratubular RAS in the entire nephron population^8^, thus contributing to renal injury and CKD^8–10^.

Ang II mediates the majority of the classical biological functions of the RAS system and regulates renal cellular and physiological responses in the renal system. Ang II is also implicated in the pathophysiological processes related to renal fibrosis via activating renal tubular cells as well as local immune cells to increase cell apoptosis and the expression of pro-fibrotic factors (e.g., TGF-β)^11^. Ang II signals through seven transmembrane-spanning G protein-coupled receptors that are characterized as AT1 or AT2 receptors based on their selective affinity for peptide and non-peptide ligands^12^. AT1R and AT2R play an important role in cell growth, differentiation and apoptosis during development^13–16^. Abnormal AT1R and AT2R expression in the renal system has also been shown to be related to kidney diseases^17,18^. However, the detailed mechanism underlying Ang II-induced renal tubular cell necroptosis and kidney injuries is not fully understood. Therefore, we hypothesized that both AT1 and AT2 receptors might modulate Ang II-induced necroptosis of renal tubular cell. In the present study, we investigated whether blocking AT1R and/or AT2R signaling can beneficially alleviate renal tubular cell necroptosis in an Ang II-infused renal damage mouse model and in Ang II-induced HK-2 cells. Our results show that AT1R and AT2R signaling might be involved in Ang II induces a receptor-interacting serine-threonine kinase 3- (RIPK3) and mixed lineage kinase domain-like (MLKL)-dependent form of regulated cell death (necroptosis) *in vitro* and *in vivo*, and these data provide novel information for the study of AT1R- and AT2R-mediated mechanisms in renal tubular cell loss and renal tubulointerstitial fibrosis.

## Materials and Methods

### *In vivo* model of Ang II-induced renal injury

The animal care and use of this study were approved by the Ethics Committee of Hainan Medical University, and the methods were carried out in accordance with the approved guidelines. Male C57BL/6 mice (8–10 weeks old) were purchased from Beijing Vital River Laboratory Animal Technology Co., Ltd. The animals were housed at an optimal temperature with a 12:12 h light-dark cycle and free access to food and water in the Hainan Research Center for drug safety evaluation.

Thirty male mice were divided randomly into five groups (n = 6 per group): an Ang II group, which received continuous Ang II infusion (1.5 µg/kg/min, Sigma) dissolved in 10% DMSO via a subcutaneous osmotic mini-pump (Alzet) after uninephrectomy surgery and was administered 0.9% sterile saline orally; Ang II + Nec-1 or losartan or PD123319 treatment groups, which received continuous Ang II infusion (1.5 µg/kg/min, Sigma) via a subcutaneous osmotic mini-pump (Alzet) after uninephrectomy surgery and were administered Nec-1 (1.65 mg/kg/day)^19,20^ (Sigma-Aldrich, USA) or losartan (10 mg/kg/day)^21^ (MedChem Express, USA) or PD123319 (10 mg/kg/day)^22^ (Cayman Chemical, USA) dissolved in 10% DMSO via intraperitoneal injection; and a control group, which was subjected to only uninephrectomy surgery and was administered 0.9% sterile saline orally. All animals were euthanized at 21 days after treatment. At this endpoint, blood samples were collected for renal function analysis. The animals were perfused with PBS, and the kidney tissues were retrieved for protein isolation and for histological analysis.

### Cell culture and stimulation

The HK-2 human renal proximal tubular epithelial cell line was purchased from ATCC (Manassas, VA, USA). The cells were cultured in DMEM/F12 medium (Gibco Life Technologies, Carlsbad, CA, USA) containing 10% FBS (HyClone, USA) and 1% penicillin and streptomycin (Beyotime, Shanghai, China) in a humidified incubator with 5% CO2 at 37 °C. After reaching 80% confluence, the cells were starved in serum-free medium for 24 h before the experiment.

Next, the cells were stimulated with Ang II at a concentration range of 10^−10^–10^−5^ M for 24 h. To elucidate the relevant mechanisms, the cells were pretreated with an AT1R antagonist (10 µM losartan^23,24^) and an AT2R antagonist (10 µM PD123319^23,24^) for 30 min or a RIP1 inhibitor (50 µM Nec-1^25^) for 30 min or a FasL inhibitor (3 µg/ml^26,27^ neutralizing human Fas ligand/TNFSF6 antibody (RD Systems, USA)) for 2 h. After pretreatment for the indicated durations, HK-2 cells were exposed to 10^−9^ M Ang II for 24 h. The HK-2 cells were exposed to 10^−9^ M Ang II for 24 h treated cells were collected at the indicated times for transmission electron microscopy (TEM), immunofluorescence staining, and Western blot analysis.

### Histopathologic and renal function analyses

A portion of the retrieved mouse kidney tissue was fixed in 4% buffered formaldehyde and embedded in paraffin. After deparaffinization and rehydration, 4-micrometer-thick sections were subjected to hematoxylin and eosin (H&E) staining. The staining results were analyzed under a bright field microscope. For quantitative analysis, at least 10 random high-power fields (400X) were selected, and the tubular damage scores were evaluated using a microscope as described by Garber *et al*.^28^.

Blood urea nitrogen and serum creatinine levels were measured using standard clinical biochemical techniques for assessing renal function in mice.

### Flow cytometric analysis

Flow cytometric analysis was performed using a FITC annexin V apoptosis detection kit (BD Biosciences, San Diego, CA) according to the manufacturer’s protocol. Briefly, cells were seeded into 6-well plates at a density of 5 × 10^5^ cells/well and exposed to Ang II at different concentrations for 24 hours. Then, the cells were harvested, washed twice with precooled PBS and resuspended in 100 µl of binding buffer. After the addition of 5 µl of FITC annexin V and PI, the cells were incubated for 15 min at room temperature in the dark, and the samples were diluted with 400 µl of binding buffer. Afterward, necrotic cells were distinguished by a flow cytometer (BD Biosciences, San Jose, CA). The percentages of the cells residing in the upper right (necrotic cells, annexin V^+^/PI^+^) regions of the annexin V-FITC scatter plots were calculated.

### Western blotting analysis

Western blotting assays were carried out as previously described^29,30^. Briefly, tissue samples or cell lysates were prepared in ice-cold radioimmunoprecipitation assay (RIPA) buffer (Beyotime, Nantong, Jiangsu, China). Equal amounts of total protein were subjected to SDS-PAGE and transferred onto PVDF membranes, which were blocked and incubated overnight with the following primary antibodies: anti-RIP3 monoclonal antibody (#95702, Cell Signaling Technologies) or anti-RIP3 polyclonal antibody (ab152130, Abcam, Cambridge, MA, USA), anti-phospho-RIP3 monoclonal antibody (ab205421, Abcam, Cambridge, MA, USA), anti-MLKL polyclonal antibody (#28640, Cell Signaling Technologies) or anti-MLKL monoclonal antibody (#14993, Cell Signaling Technologies), anti-phospho-MLKL monoclonal antibody (ab208910, Abcam, Cambridge, MA, USA) or anti-phospho-MLKL monoclonal antibody (#91689, Cell Signaling Technologies), or anti-β-actin monoclonal antibody (sc-47778, Santa Cruz Biotechnology, CA, USA). After washing, the membranes were incubated with the appropriate secondary antibodies conjugated with horseradish peroxidase (HRP). Immune-reactive signals were visualized by an enhanced chemiluminescence (ECL) kit (Millipore, USA) on a Syngene PXi6 Access imaging system (Frederick, MD). The band intensities were quantified using Image-Pro Plus 6.0.

### TEM

Kidney tissue and cells treated with the reagents described previously were harvested; 1-mm^3^ renal tissue fragments and centrifuged cells were fixed in 4% glutaraldehyde phosphate buffer (pH 7.4) overnight at 4 °C, rinsed with PBS and postfixed in 2% osmium tetroxide. Then, the fixed renal tissue fragments and cells were dehydrated in an ascending series of ethanol and embedded in epoxy resin. Finally, ultrathin sections (60–70 nm) were stained with uranyl acetate and alkaline lead citrate, and the ultrastructure of the cells was visualized under a transmission electron microscope (Hitachi-7700, Japan).

### Immunofluorescence detection of RIP-3 and ***in situ*** fluorescent TUNEL staining

Sagittal kidney tissue sections (4-µm-thick) and HK-2 cells seeded on chamber slides (Thermo Scientific, USA) and incubated with the previously described treatments were prepared for RIP3 immunofluorescence staining and *in situ* fluorescent TUNEL staining. First, the sections and cells were fixed with 4% paraformaldehyde (Sigma-Aldrich, USA), followed by permeabilization in 0.1% Triton X−100 and incubation with 5% BSA (Sigma-Aldrich, USA). The slides and cells were incubated with an anti-RIP3 monoclonal antibody (#95702, Cell Signaling Technologies, Danvers, MA, USA) or anti-RIP3 polyclonal antibody (ab152130, Abcam, Cambridge, MA, USA) overnight at 4 °C and then with Alexa Fluor 594-conjugated goat anti-rabbit IgG (H + L) (#4413, Cell Signaling Technologies). After rinsing 3 times with 0.1 M PBS (pH 7.4), the samples were incubated with *in situ* cell death detection kit reagents (Fluorescein, Roche, Basel, Switzerland) according to the manufacturer’s instructions and counterstained with 4′,6-diamidino-2-phenylindole (DAPI). Finally, the images were captured by confocal microscopy (LEICA TCS SP2, Wetzlar, Germany), and cell counting was performed by a pathologist blinded to the experimental conditions.

### Statistical analysis

The data are presented as the means ± SEM. Multiple group comparisons were performed by one-way ANOVA followed by the Bonferroni procedure for the comparison of means. p < 0.05 was considered statistically significant.

## Results

### Inhibition of AT1R and AT2R suppresses renal tubular epithelial cell necroptosis in Ang II-treated renal injury mice

**Figure 1:**
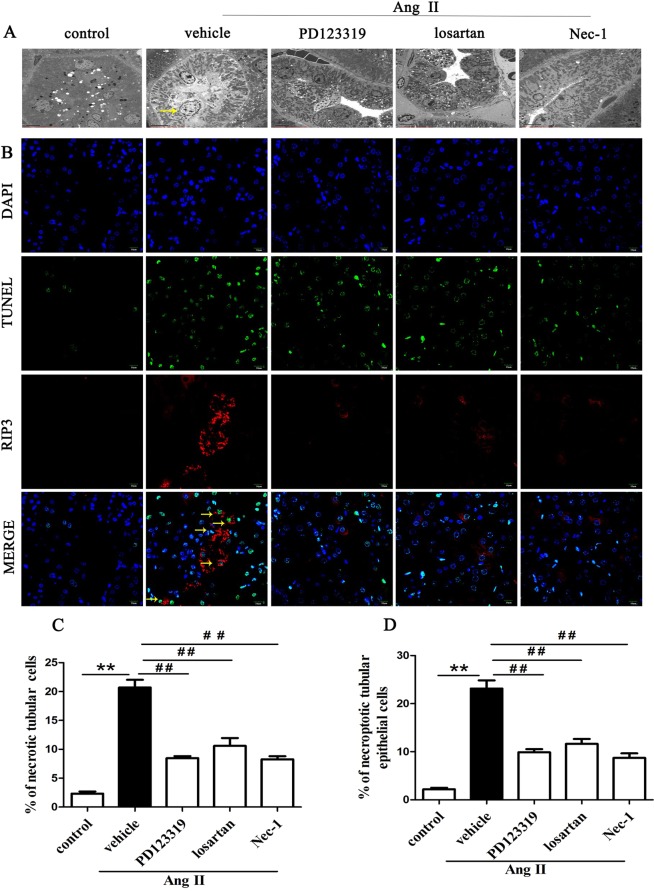
Inhibition of AT1R and AT2R suppresses renal tubular epithelial cell necroptosis in Ang II-treated renal injury mice. (**A**) Representative TEM images of necrotic tubular epithelial cells (as shown by the arrow) in the kidney tissues of Ang II-treated mice. (**B**) TUNEL-stained (green fluorescence) cells in the mouse renal tubules were costained to detect RIP3 (red fluorescence) and the nuclei (DAPI, blue fluorescence). The scale bars represent 10 μm. (**C**) The % ratio of necrotic tubular epithelial cells (as shown by the arrow) was analyzed. (**D**) The data are presented as the % ratio of TUNEL-positive and RIP3-positive tubular cells (necroptotic tubular cells). For the data in (**C, D**), the values are reported as the means ± S.E.M. of n = 6; **p < 0.01, versus control; ^#^p < 0.05, ^##^p < 0.01, versus the Ang II-treated group. Ang II: angiotensin II.

We explored whether blocking AT1R and AT2R would affect renal tubular epithelial cell necrosis using TEM. Figure 1A shows the microstructural changes observed in renal tubular epithelial cells under TEM. In the Ang II + vehicle group, many renal tubular epithelial cells showed a necrotic morphology, with markedly swollen cells, membranolysis and organelle contents disappearance and extensive intracellular vacuole formation. These findings are consistent with the typical morphological features of necroptotic cell death^20,31^. In addition, apoptotic renal tubular epithelial cells were occasionally observed in kidneys derived from Ang II- infused renal injury mice. However, the integrity of renal tubular epithelial cells were preserved in the Ang II + Nec-1 treated mice, and the percentage of necrotic renal tubular epithelial cells in the Ang II + Nec-1 treated mice was lower than that in the Ang II + vehicle group (p < 0.01) (Fig. 1A,C). Importantly, we observed that the addition of losartan or PD123319 also preserved the integrity of renal tubular epithelial cells (p < 0.01) (Fig. 1A,C), which was similar to the effects of Nec-1 (p > 0.05) (Fig. 1A,C).

To confirm that the necrotic renal tubular epithelial cells under TEM were necroptotic cells, we performed RIP3 immunofluorescence staining and *in situ* fluorescent TUNEL staining. Rasl-Kraupp B *et al*. and other researchers reported that necrosis can also generate DNA fragments that react with the TUNEL reaction solution^31,32^. Therefore, TUNEL staining and RIP3 immunofluorescence staining are used to determine the type of cell death that a cell undergoes. Cells that were positive for TUNEL staining and positive for RIP3 were considered to be necroptotic cells in this study. As shown in Fig. 1B,D, we found that Ang II treatment significantly increased TUNEL-positive and RIP3-positive expression in tubular epithelial cells compared with the control treatment (p < 0.01). However, this TUNEL-positive and RIP3-positive expression was significantly subdued in the Nec-1-treated mice with chronic Ang II infusion (p < 0.01) (Fig. 1B,D), further suggesting that necroptosis is involved in renal tubular epithelial cell necrosis. Importantly, we also found that losartan- or PD123319-treated mice had significantly lower percentages of TUNEL-positive and RIP3-positive tubular epithelial cells in their kidney tissues than Ang II-infused mice (p < 0.01) (Fig. 1B,D). These data suggest that blocking AT1R with losartan and AT2R with PD123319 might supressed renal tubular epithelial cell necroptosis in chronic Ang II-infused mice.

### Inhibition of AT1R and AT2R diminishes necroptosis-related proteins in Ang II-induced renal injury mice

RIP3, MLKL and their phosphorylated proteins are the key proteins of the necroptosis pathway, and their expression levels have been shown to correlate closely with necroptosis^33^. RIP3 is activated by binding and interacting with RIP1, and activated RIP3 recruits and subsequently phosphorylates downstream signaling molecule MLKL (on Ser345/Ser347 in mouse MLKL). MLKL phosphorylation is believed to trigger a molecular switch for programmed cell death, and result in necroptosis^34^. Therefore, we assessed RIP3, MLKL and their phosphorylated proteins expression in kidney tissues from Ang II-infused mice using Western blotting.

**Figure 2:**
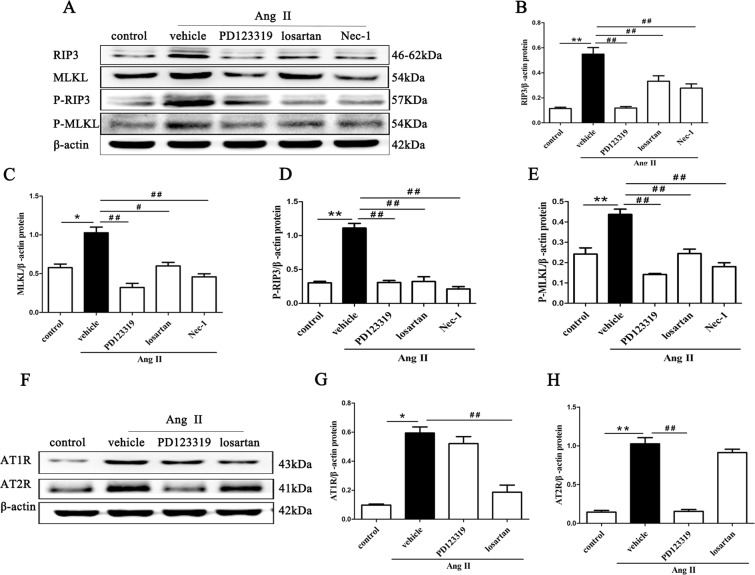
Inhibition of AT1R and AT2R diminishes necroptosis-related proteins in Ang II-induced renal injury mice. Tissue lysates of the retrieved mouse kidneys were subjected to SDS-PAGE and Western blotting with RIP3, p-RIP3, MLKL and p-MLKL (**A**), AT1R and AT2R (**F**) antibodies and a β-actin antibody. The intensities of the bands were determined quantitatively using Image-Pro Plus 6.0 (**B–E,G,H**). For the data in (**B–E,G,H**), the values are reported as the means ± S.E.M. of n = 6; *p < 0.05 and **p < 0.01, versus control; ^#^p < 0.05, ^##^p < 0.01, versus the Ang II-treated group. Ang II: angiotensin II.

The Western blotting results revealed that Ang II treatment significantly up-regulated the protein expression of the necroptotic cell markers RIP3, p-RIP3 and MLKL, p-MLKL (p < 0.01) (Fig. 2A–E). However, blocking AT1R activity with losartan or inhibiting AT2R activity with PD123319 effectively suppressed the Ang II-induced expression of these proteins (p < 0.01) (Fig. 2A–E), which was similar to the effects of Nec-1 (p > 0.05) (Fig. 2A–E).

In addition, the expression levels of AT1R and AT2R were significantly higher in kidney tissues from Ang II-infused mice than from the control group (p < 0.01) (Fig. 2F–H). Losartan reduced the protein expression of AT1R (p < 0.01) but had no effect on AT2R (p > 0.05). PD123319 also diminished AT2R levels but did not change AT1R levels (p > 0.05).

These results suggest that AT1R and AT2R might respectively modulate the RIPK3-MLKL-mediated necroptosis induced by Ang II in Ang II-infused mice.

### Inhibition of Necroptosis improves renal function and renal injury in a mouse model of Ang II-induced renal injury

**Figure 3:**
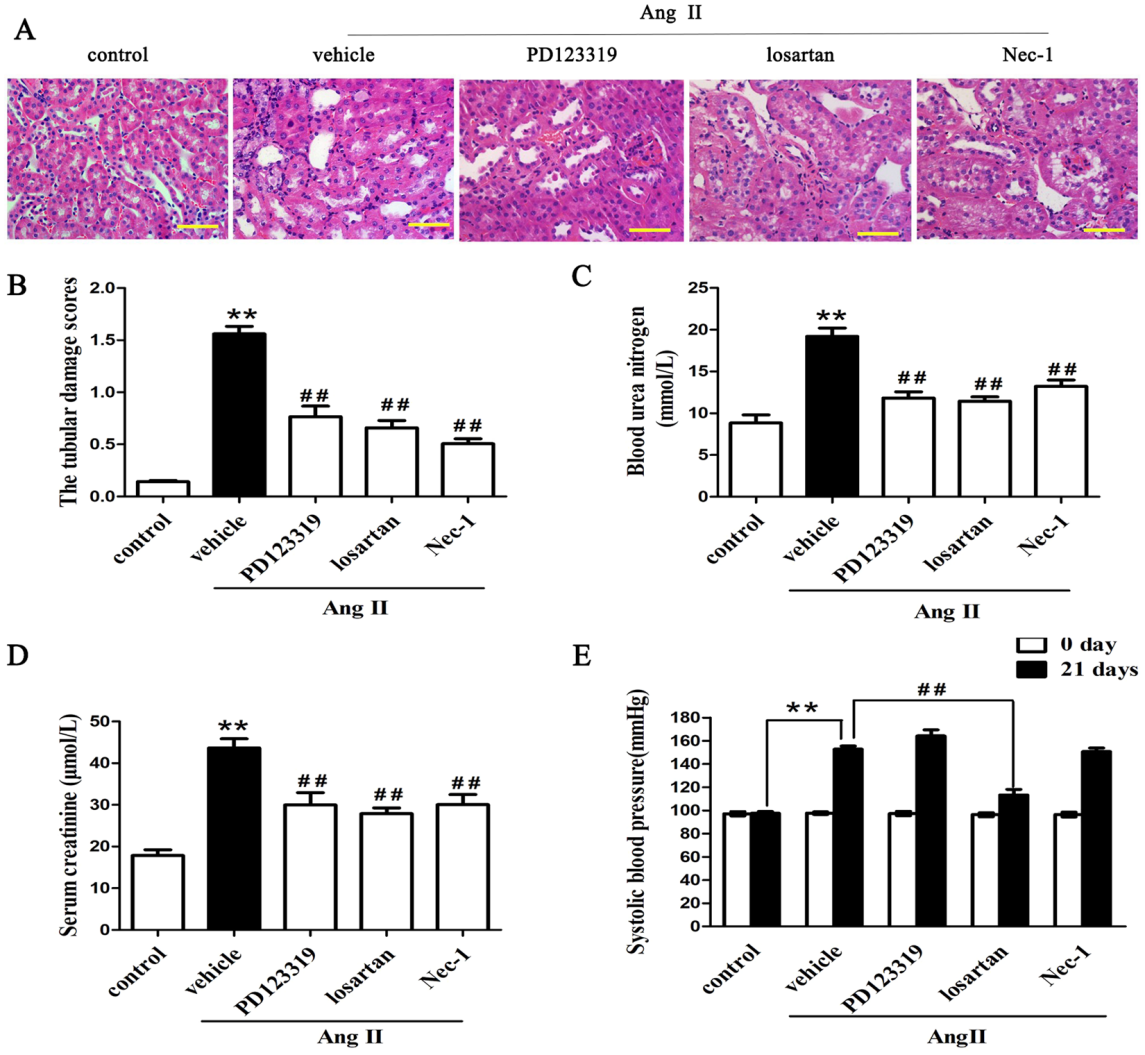
Necroptosis inhibition improves renal function and renal injury in a mouse model of Ang II-induced renal injury. (**A**) H&E staining of the kidney tissues retrieved from Ang II-infused mice treated with or without PD123319 or losartan or Nec-1 (n = 6 per group). Representative images are shown. (**B**) The tubular damage scores were assessed using a light microscope. Ang II-induced increases in blood urea nitrogen (**C**) and serum creatinine (**D**) levels were significantly reversed by PD123319 or losartan or Nec-1. (**E**) The systolic blood pressure was assessed. **p < 0.01 versus control; ^##^p < 0.01 versus the Ang II-treated group. Ang II: angiotensin II.

We next examined the histopathologic features of kidney tissues from Ang II-induced renal injury mice to determine the effect of necroptosis on renal pathologic structure. H&E staining revealed that tubular injury scores were significantly higher in the Ang II-infused group than in the control group (p < 0.01) (Fig. 3A,B). However, this renal injury response was significantly attenuated in the Nec-1-treated, PD123319-treated and losartan-treated mice with chronic Ang II infusion (p < 0.01) (Fig. 3A,B).

We also examined serum creatinine and blood urea nitrogen levels as markers of renal function. As shown in Fig. 3C,D, serum creatinine and blood urea nitrogen levels were significantly higher in the Ang II-infused group than in the control group (p < 0.01). However, the Ang II-induced increases in serum creatinine and blood urea nitrogen levels were significantly mitigated by Nec-1, losartan and PD123319. In addition, we found that Ang II infusion increased systolic blood pressure in AngII-treated mice (p < 0.01). Blocking AT1R with losartan significantly mitigated increased blood pressure, but Nec-1 and PD123319 had no remarkable effect on the increased blood pressure of the AngII-infused mice (Fig. 3E). The data suggested that ANG II-induced necroptosis might be involved both AT1 and AT2 receptors, and blockade of both may beneficially alleviate renal tubular epithelial cells necroptosis and renal tubule injury in Ang II-induced renal injury mice.

### Ang II induces RIPK3-MLKL-mediated necroptosis in HK-2 cells

**Figure 4:**
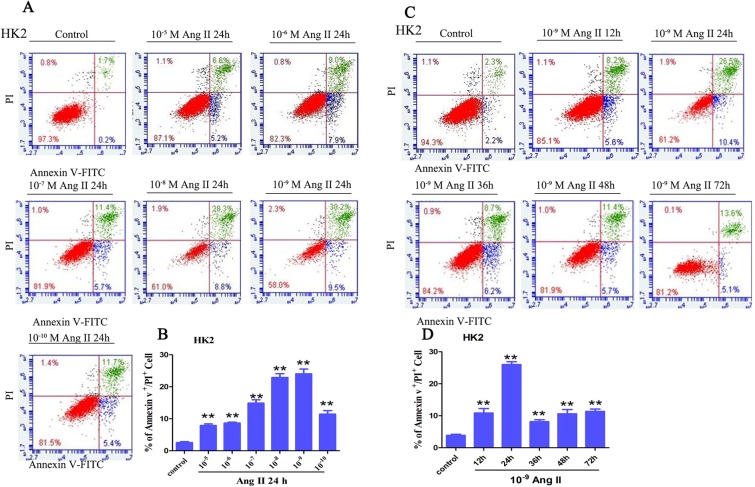
Detection of necrotic tubular epithelial cells using a flow cytometer. (**A**,**B**) HK-2 cells were treated with varying concentrations of Ang II (10^−10^–10^−5^ M) for 24 h. (**C**,**D**) HK-2 cells were exposed to 10^−9^ M Ang II for different times. After treatment, HK-2 (upper panel) cells were stained with annexin V-FITC and PI to determine cell necrosis (annexin V^+^/PI^+^ cells) using a flow cytometry assay. The bar chart shows that the ratio of annexin V^+^/PI^+^ cell numbers was highest in the HK-2 cells treated with 10^−9^ M Ang II for 24 h.

HK-2 cells were subjected to different concentrations of Ang II (10^−5^-10^−10^ M) for 24 h. When the Ang II concentration was 10^−8^ M, the ratio of annexin V^+^/PI^+^ cells was remarkably increased (p* < *0.01, Fig. 4A,B), and the ratio of annexin V^+^/PI^+^ cells was highest in the HK-2 cells treated with 10^−9^ M Ang II (p* < *0.01, Fig. 4A,B). In addition, cells were pretreated with 10^−9^ M Ang II for various times (0, 12, 24, 48, 36, and 72 h), and remarkable increases in the annexin V^+^/PI^+^ cell ratio were observed after 24 h of Ang II stimulation (p* < *0.01, Fig. 4C,D). Taken together, these data indicated that Ang II induces necrosis in HK-2 cells in a dose- and time-dependent manner, and 10^−9^ M and 24 h were selected as the Ang II stimulation conditions for the subsequent experiments.

**Figure 5:**
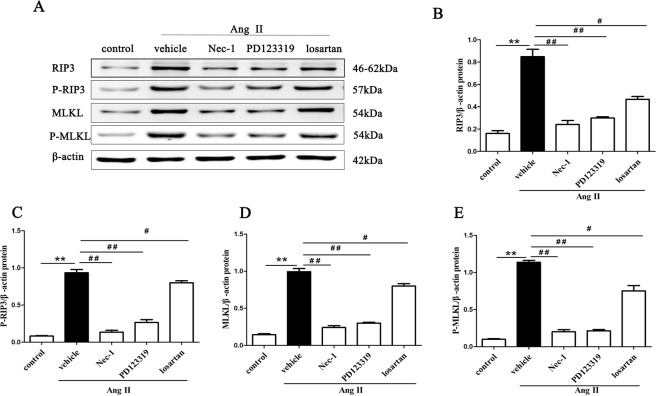
Ang II induces RIPK3-MLKL-mediated necroptosis in HK-2 cells. HK-2 cells were pretreated with or without 10 µM losartan and 10 µM PD123319 for 30 min or 50 µM Nec-1 for 30 min, followed by exposure to 10^−9^ M Ang II for 24 h. Representative Representative blots (**A**) and Western blot analysis (**B–E**) for necroptosis marker proteins: RIP3, MLKL, p-RIP3, and p-MLKL antibodies were used; β-actin was used as a loading control. The results are representative of three independent experiments. The intensities of the bands were determined quantitatively using Image-Pro Plus 6.0 **p < 0.01 versus control; ^#^p < 0.05, ^##^p < 0.01 versus the Ang II-induced group. Ang II: angiotensin II.

To test whether Ang II-induced necrosis in HK-2 cells might be RIPK3 -MLKL-mediated necroptosis, we also investigated the effects of Ang II on key necroptosis proteins (RIP3, MLKL, P-RIP3, and P-MLKL) using Western blotting (Fig. 5). Compared with cells stimulated with PBS alone, HK-2 cells stimulated with Ang II had significantly higher expression levels of RIP3, MLKL, p-RIP3 and p-MLKL (p < 0.01) (Fig. 5A–E), and these changes were blocked effectively by Nec-1, PD123319 and losartan (p < 0.01). Interestingly, a quantitative analysis confirmed that the RIP3, MLKL, p-RIP3 and p-MLKL protein levels were similar among the three pretreatment groups (p > 0.05). Collectively, these results suggest that RIPK3-MLKL-mediated necroptosis may be at least in part responsible for Ang II-induced necrosis in HK-2 cells, and both AT1 and AT2 exert important effect on necroptosis of Ang II-induced HK-2 cells.

### Inhibition of AT1R and AT2R diminishes Ang II-induced necroptosis in HK-2 cells

**Figure 6:**
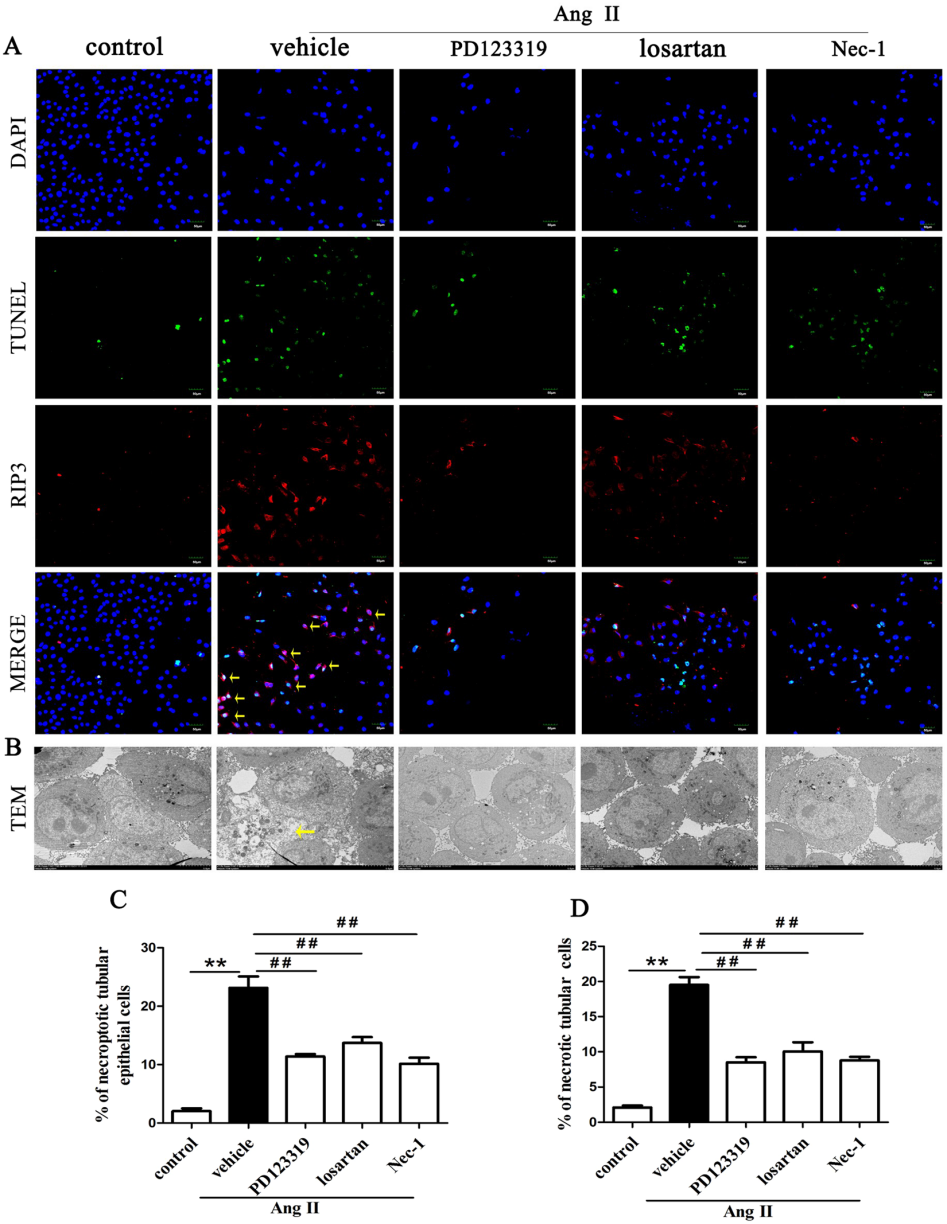
Inhibition of AT1R and AT2R diminishes Ang II-induced necroptotic HK-2 cells. (**A**) TUNEL-stained (green fluorescence) HK-2 cells treated with 10^−9^ M Ang II for 24 h were costained to detect RIP3 (red fluorescence) and the nuclei (DAPI, blue fluorescence). The scale bars represent 50 μm. (**B**) Representative TEM images of necroptotic HK-2 cells (as shown by the arrow) after treatment with 10^−9^ M Ang II for 24 h. The scale bars represent 5 μm. **(C)** The data are presented as the % ratio of necroptotic HK-2 cells(as shown by the arrow). **(D)** The data are presented as the % ratio of necrotic cells among all HK-2 cells treated with 10^−9^ M Ang II for 24 h. For the data in (**C, D**), the values are reported as the means ± S.E.M. of n = 6; **p < 0.01, versus control; ^##^p < 0.01, versus the Ang II-treated group. Ang II: angiotensin II.

HK-2 cells were cultured *in vitro* and analyzed with fluorescent TUNEL staining and RIP3 immunostaining assays to further investigate the role of AT1R and AT2R in Ang II-induced HK-2 cell death (Fig. 6A). The quantitative analysis of fluorescent TUNEL staining and RIP3 immunostaining revealed that the necroptotic incidence was higher in HK-2 cells treated with Ang II than in cells stimulated with PBS alone (p < 0.01, Fig. 6A,C), whereas the cells treated with PD123319, losartan or Nec-1 had a significantly lower necroptotic incidence than cells treated with Ang II (p < 0.01, Fig. 6A,C).

TEM was used to observe the ultrastructure of HK-2 cells stimulated with Ang II to better understand their morphological characteristics. The TEM results demonstrated that all of the HK-2 cells stimulated with Ang II displayed a necrotic morphology (Fig. 6B). More importantly, cotreatment with Ang II and losartan or PD123319 greatly reduced the necrotic incidence of HK-2 cells (Fig. 6B,D), which was similar to the decreased necrotic incidence of HK-2 cells treated with Nec-1 (p > 0.05). These results were consistent with those of the fluorescent TUNEL staining and RIP3 immunostaining assays.

**Figure 7:**
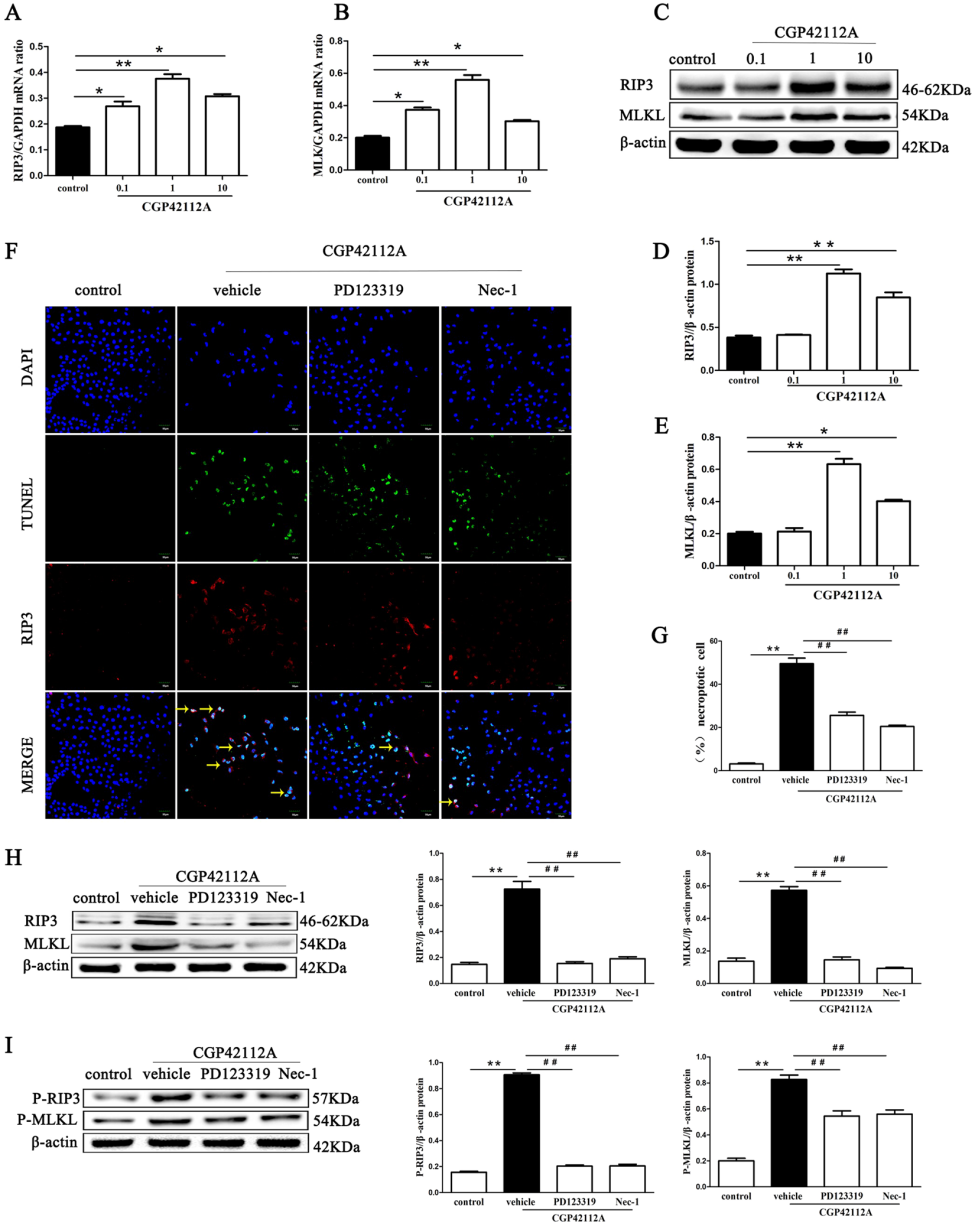
The AT2 agonist CGP42112A facilitates necroptosis of HK-2 cells. Effects of CGP42112A on HK-2 cells necroptosis were determined. HK-2 cells stimulated with CGP42112A at a concentration of 0, 0.1, 1, and 10µmol/L for 24 h. Then, qPCR and Western Blot analysis was performed to detect the level of RIP3 and MLKL mRNA and protein (**A**–**E**). Next, HK-2 cells were pretreated with an AT2R antagonist (10 µM PD123319) and a RIP1 inhibitor (50 µM Nec-1) for 30 min, then the cells were treated with 0.1 µM CGP42112A for 24 h. **F** shows representative images of immunofluorescence staining for RIP3 (red fluorescence) and *in situ* fluorescence TUNEL staining (green fluorescence). Scale bars represent 50 μm. (**G**) The data are presented as the % ratio of necroptotic HK-2 cells (TUNEL-positive and RIP3-positive cells). Representative blots (**H**) and Western blot analysis (**I**) for RIP3, MLKL, p-RIP3, and p-MLKL proteins; β-actin was used as a loading control. The results shown are representative of three independent experiments. *p < 0.05 versus control; **p < 0.01 versus control; ^##^p < 0.01 versus the CGP42112A group.

To prove AT2R might function as critical mediators in necroptosis of renal tubular cell, HK-2 cells were treated with the AT2 agonist CGP42112A (MedChemExpress, America) at different concentrations (0, 0.1, 1, and 10 µmol/L) for 24 hour. In qPCR and Western Blot analysis, the level of RIP3 and MLKL mRNA and protein was at the highest when the cells were treated with 1 µmol/L CGP42112A (Fig. 7A–E). It is more important that we found CGP42112A (1µmol/L) significantly increased the percentage of necroptotic HK-2 cells (p < 0.01) (Fig. 7F–G) with fluorescent TUNEL staining and RIP3 immunostaining assays, which was blocked by PD123319 or Nec-1. In addition, we investigated the effects of CGP42112A on RIP3, MLKL, P-RIP3, and P-MLKL using Western blotting, and found that stimulation with CGP42112A increased significantly levels of RIP3, MLKL, p-RIP3 and p-MLKL protein in HK-2 cells (p < 0.01) (Fig. 7H,I), and the increase was blocked effectively by PD123319 and Nec-1 (p < 0.01).

### Inhibition of AT1R and AT2R mitigates the expression of Fas/FasL signaling molecules in Ang II-induced HK-2 cells

**Figure 8:**
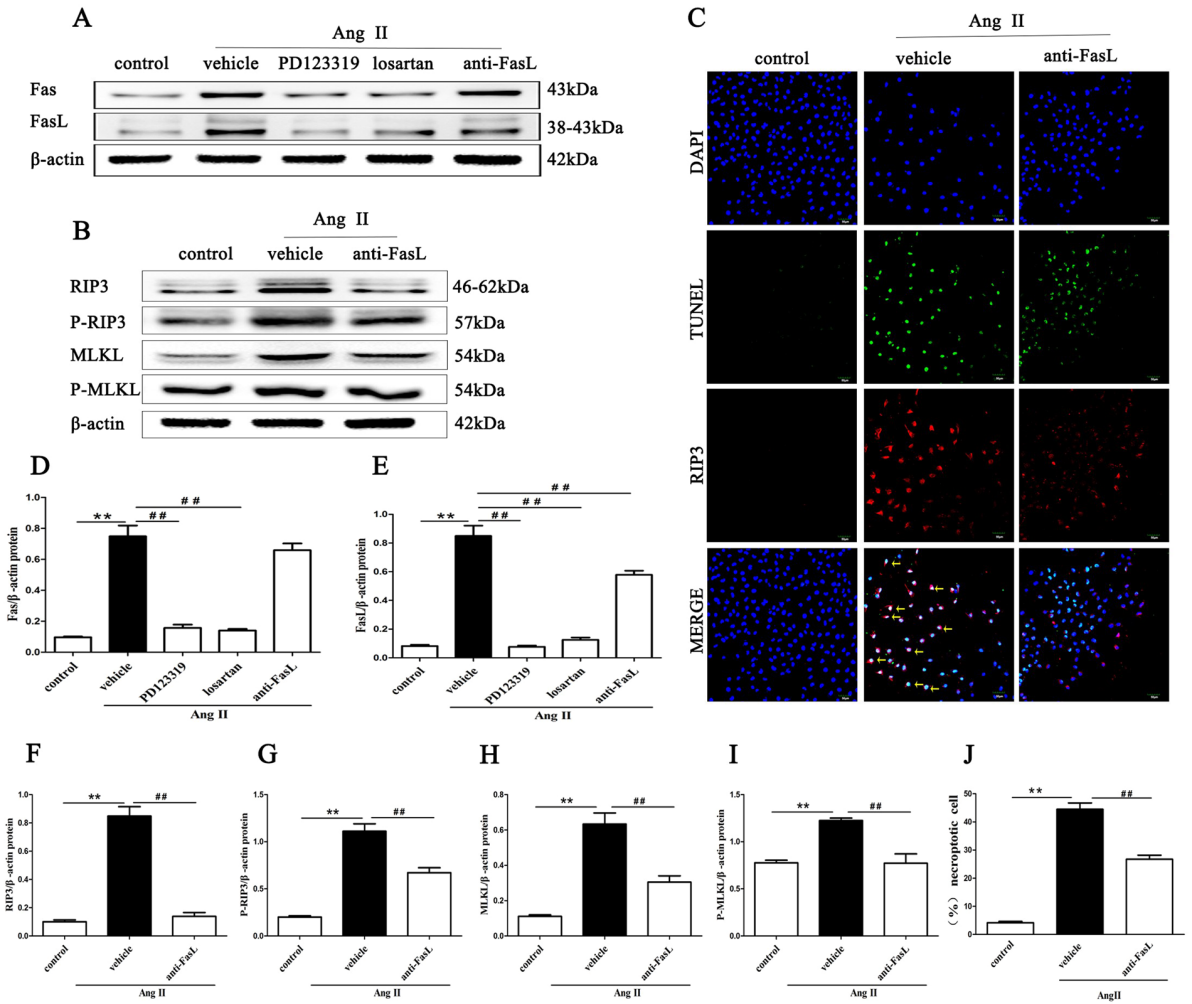
Inhibition of AT1R and AT2R mitigates the expression of Fas/FasL signaling molecules in Ang II-induced HK-2 cells. Effects of FasL blockade via neutralizing human Fas ligand/TNFSF6 antibody on Ang II-induced necroptosis in HK-2 cells were determined. HK-2 cells were pretreated with 3 µg/ml neutralizing human Fas ligand/TNFSF6 antibody for 2 h and exposed to 10^−9^ M Ang II for 24 h. Then, Western blotting was performed to detect Fas and FasL (**A,D,E**), RIP3, MLKL, p-RIP3, and p-MLKL (**B,F–I**) levels and β-actin was used as a loading control. **C** shows representative images of immunofluorescence staining for RIP3 (red fluorescence) and *in situ* fluorescence TUNEL staining (green fluorescence). Scale bars represent 50 μm. **(J)** The data are presented as the % ratio of necroptotic HK-2 cells (TUNEL-positive and RIP3-positive cells). The results shown are representative of three independent experiments. The intensities of the bands were determined quantitatively using Image-Pro Plus 6.0 **p < 0.01 versus control; ^##^p < 0.01 versus the Ang II-induced group. Ang II: angiotensin II.

Fas and FasL protein expression was detected in Ang II-stimulated HK-2 cells by Western blotting. Fas and FasL expression levels were significantly higher in HK-2 cells stimulated with Ang II than in cells stimulated with PBS alone (p < 0.01, Fig. 8A,D,E). Losartan and PD123319 effectively mitigated the Ang II-induced increases in Fas and FasL signaling molecule expression (p < 0.01). Importantly, disruption of FasL significantly suppressed the necroptotic incidence (p < 0.01, Fig. 8C,J) and necroptosis-related proteins, including RIP3, MLKL, P-RIP3 and P-MLKL and FasL expression (p < 0.01, Fig. 8B,F–I). These results suggest that AT1R and AT2R and the subsequent Fas/FasL signaling might be involved in Ang II-induced necroptosis.

## Discussion

The abnormal activation of Ang II plays a central role in the initiation and progression of CKD^35–38^, and the renoprotective effects of inhibiting its receptors are well known. It has been increasingly recognized that in addition to its hemodynamic effects, Ang II can exert direct effects on kidney cells^38–41^. Our current study revealed a novel paradigm for explaining the mechanisms of action of Ang II. We selected an Ang II infusion mouse model, wherein we provided a constant supply of Ang II to the tissue with an osmotic pump. The model used in the present study allowed us to determine the direct effect of Ang II on renal tubular cells without involving the role of ACE in Ang II production. We used TEM and confocal microscopy to find that Ang II effectively increased the percentage of necroptotic tubular epithelial cells compared with apoptosis in Ang II-infused renal injury mice and in Ang II-stimulated HK-2 cells. In accordance with these findings, the protein expression levels of the necroptotic cell markers RIP3, MLKL, p-RIP3 and p-MLKL were significantly increased by Ang II treatment *in vivo* and *in vitro*. These results were notably different from those of previous reports showing that Ang II can induce apoptosis in renal proximal tubular cells via both AT1R and AT2R^42^, suggesting that Ang II signals can have detrimental effects on renal tubular cells via necroptosis pathway.

Classically, the majority of biological actions of Ang II are mediated by its cognate AT1 G protein-coupled receptor. AT1R has been implicated in a variety of pathologic conditions, such as hypertension, chronic heart failure and diabetic nephropathy. In addition, AT1R has been implicated in a variety of pathologic conditions, such as hypertension, cell growth, differentiation during development^13–16^ and apoptosis in renal proximal tubular cells. In the present study, we investigated the effects of AT1R on Ang II-induced necroptosis in renal tubular cells. The results showed that Ang II treatment significantly upregulated the expression of AT1R. Importantly, antagonists of AT1R activity, such as losartan, could effectively suppress the percentage of necroptotic renal tubular epithelial cells and the expression of necroptosis-related proteins *in vivo in vitro*. Therefore, we speculate that AT1R signaling might be mediated partly Ang II-induced renal tubular cell necroptosis.

AT2R plays an important role in cell growth, differentiation and apoptosis during development^14,15^, but the pathophysiological role of AT2R in CKD has not been defined clearly^43–45^. A previous study showed that there was reciprocal expression between AT1R and AT2R in response to Ang II infusion, with evident AT1R upregulation and AT2R downregulation^46,47^. However, the current data clearly challenge that concept. At least in kidney tissue, Ang II-treated adult mice exhibited higher AT2R protein levels with increasing AT1R levels. Importantly, functionally antagonizing AT2R activity with PD123319 could effectively diminish renal tubular epithelial cell necroptosis induced by Ang II cytotoxicity. Furthermore, we found that CGP42112A (an AT2 agonist) significantly increased the level of RIP3 and MLKL in HK-2 cells and the percentage of necroptotic HK-2 cells, which was blocked by PD123319 or Nec-1. These results suggest that both AT1 and AT2 receptors might be involved in Ang II-induced necroptosis of renal tubular cell, which is similar to Ang II-induced apoptosis occurring via both AT1R and AT2R^42^.

Lastly, our results indicate that blocking AT1R and AT2R signaling effectively diminishes the expression of the death receptors Fas and FasL in the kidney. Fas and its ligand (FasL) are members of the tumor necrosis factor receptor superfamily and induce a series of intracellular signaling events, culminating in the activation of death-inducing signaling complexes, which promote apoptosis^42,48^. In this study, we also observed that blocking FasL significantly suppressed necroptosis in renal tubular epithelial cells and necroptosis-related proteins, suggesting that Fas/FasL, as subsequent signaling molecules of AT1R and AT2R signaling, might be involve in Ang II-induced necroptosis of renal tubular cell.

In summary, Ang II-induced necroptosis in renal tubular cells is a novel redundant mechanism of Ang II-mediated renal tubular injury and CKD, and blocking AT1R and AT2R effectively mitigates Ang II-induced increases in necroptosis of renal tubular cells. Therefore, blocking AT1R and AT2R may be a novel therapeutic approach for alleviating the excessive loss of renal tubular cells in the progression of CKD.
